# Supplementary figures and images for: A Nuclear Magnetic Resonance Spectroscopy Method in Characterization of Blood Metabolomics for Alzheimer’s Disease
Source: Metabolites. 2022 Feb 15;12(2):181. doi: 10.3390/metabo12020181 (PMC8878886; doi:10.3390/metabo12020181)

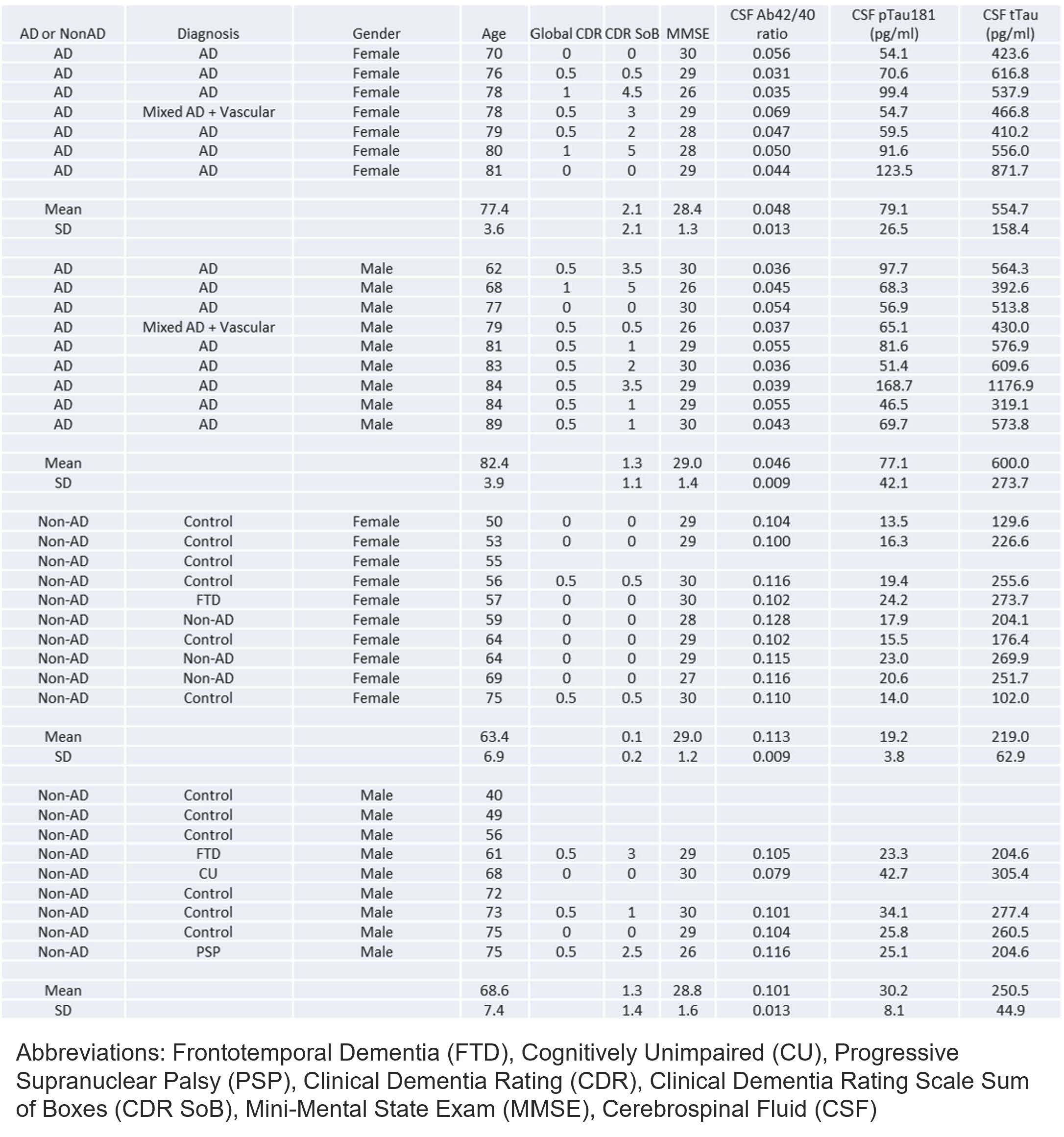

Supplement: Supplementary file 1 [file metabolites-12-00181-s001.zip › Table S1.jpg]

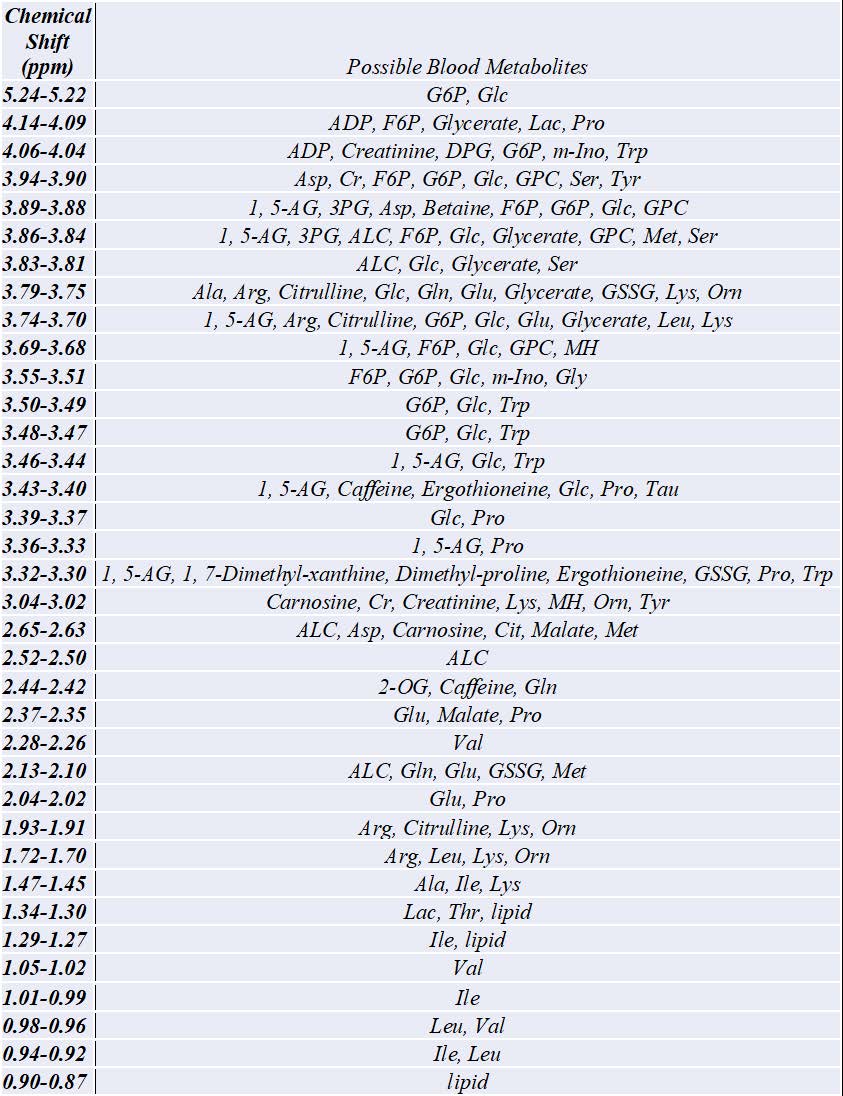

Supplement: Supplementary file 1 [file metabolites-12-00181-s001.zip › Table S2.jpg]

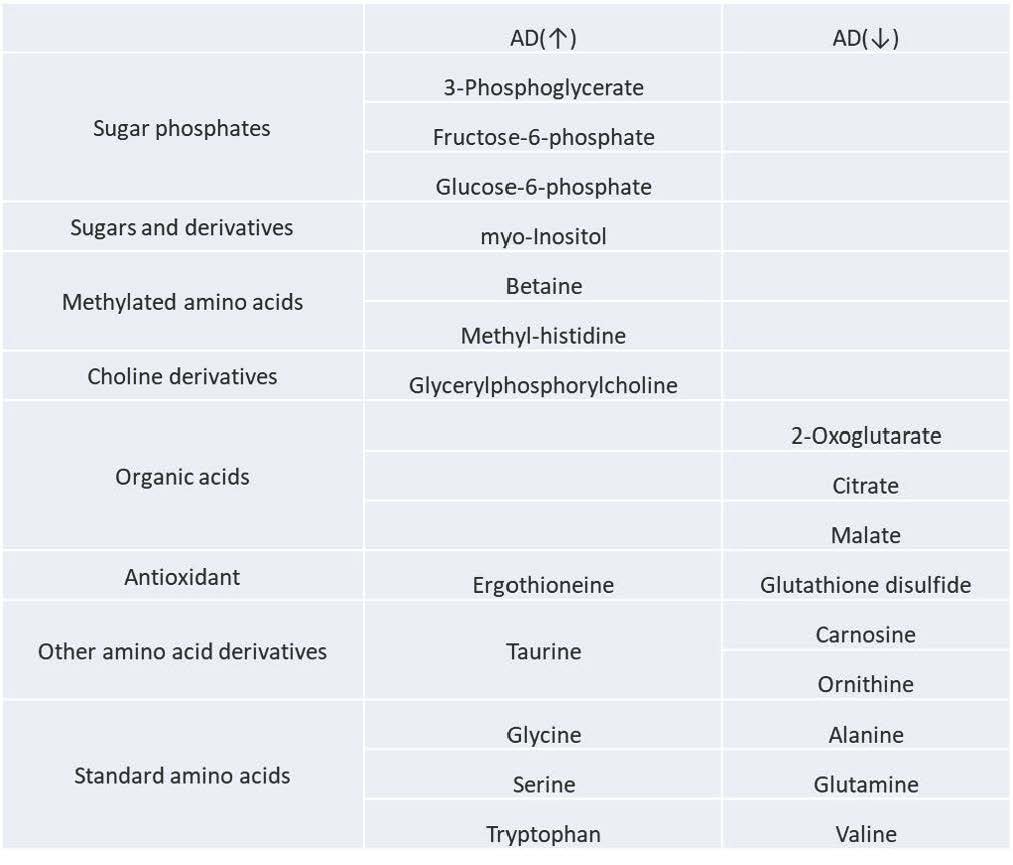

Supplement: Supplementary file 1 [file metabolites-12-00181-s001.zip › Table S3.jpg]
